# Rigosertib inhibits MEK1–ERK pathway and alleviates lipopolysaccharide‐induced sepsis

**DOI:** 10.1002/iid3.458

**Published:** 2021-06-01

**Authors:** Yin Wang, Pengfei Du, Donghui Jiang

**Affiliations:** ^1^ Department of Intensive Medicine The Affiliated Hospital of Jiangnan University Wuxi Jiangsu China

**Keywords:** LPS, MEK1/ERK, rigosertib, sepsis

## Abstract

**Background:**

Here, by using the lipopolysaccharide (LPS)‐induced mice sepsis model, we treated septic wild‐type (WT) mice or MEK1^DD^ mice with rigosertib to evaluate its prospective effects on sepsis.

**Methods:**

We also generated macrophages derived from bone marrow from WT or MEK1^DD^ mice. These macrophages were pretreated with rigosertib and then induced with LPS or poly I:C.

**Results:**

Rigosertib suppressed LPS or poly I:C‐induced expression of inflammatory cytokines (tumor necrosis factor‐alpha [TNF‐α] and interleukin‐6 [IL‐6], and IL‐23) in WT bone marrow–derived macrophages while failed to affect the upregulation of TNF‐α and IL‐6 in LPS‐treated bone marrow–derived macrophages from MEK1^DD^ mice. Rigosertib promoted survival rate, ameliorated lung injury, and reduced inflammatory cytokine levels in serum of WT septic mice.

**Conclusion:**

In contrast, the effects of rigosertib on sepsis were abrogated in septic MEK1^DD^ mice, which had inducible constitutive activation of MEK1 signaling. Rigosertib alleviated LPS‐induced sepsis inhibits MEK1/ERK signaling pathway.

## Graphical abstract

Rigosertib promoted survival rate, ameliorated lung injury, and decreased tumor necrosis factor‐alpha (TNF‐α) and interleukin‐6 (IL‐6); and IL‐1β levels. Rigosertib alleviated lipopolysaccharide‐induced sepsis inhibits MEK1/ERK signaling pathway.

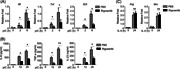

## 1 INTRODUCTION

Sepsis is an inflammatory disease with great morbidity, which causes many deaths every year.^1^ Sepsis is one of the major clinical problems and causes severe economic burden.^2^

Bacterial infection‐induced inflammation is the key factor in sepsis. Immune dysfunction devotes greatly to sepsis development. In early sepsis, the pattern recognition receptors including toll‐like receptors (TLRs) are activated by pathogen‐associated molecular patterns (PAMPs), which promote innate immune responses.^3^ The following intracellular signaling process leads to the expression of inflammation‐related genes. In sepsis, the stimulation which is recognized by the immune system is far greater than in regular infection, resulting in a cytokine storm. This dysregulated hyperinflammation leads to many symptoms in early sepsis. Macrophages, which are the major cells to produce proinflammatory cytokines in sepsis, play a central role in sepsis pathophysiology.^4^, ^5^

The mitogen‐activated protein kinase (MAPK) family consists of c‐Jun N‐terminal kinases (JNKs), extracellular signal‐regulated kinase 1/2 (ERK1/2), and p38 MAPKs. These kinases are major downstream targets of TLRs and other PRRs responsible for the induction of inflammatory signaling.^6^ ERK1/2 are activated by the MAPK/ERK kinase‐1/2 (MEK1/2). Once activated, ERK1/2 are able to phosphorylate many other kinases and transcription factors, resulting in the production of inflammation factors including tumor necrosis factor‐alpha (TNF‐α).^7^ Studies suggested that MEK/ERK signaling pathway is a therapeutic target to prevent inflammation and sepsis. For example, Shi‐Lin et al. showed that inhibition of MEK/ERK decreased the circulating TNF‐α level and prevented mortality in septic mice.^8^

Rigosertib is an anticancer agent which could cause mitotic arrest and induce apoptosis in many cancer cells but does not affect normal cells.^9^ It has been shown that rigosertib interacts with the RAS‐binding domain of RAF, which prevents RAF binding to RAS, inhibits RAF activation, and finally leads to suppression of the RAS–RAF–MEK–ERK signaling pathway.^10^ Rigosertib have also been shown to suppress inflammation.^11^ Furthermore, RAS–RAF–MEK–ERK pathway are activated through TLR engagement.^12^, ^13^ Taken together, these findings suggest potential roles of rigosertib in sepsis. In the current study, the effects of rigosertib on sepsis were evaluated in lipopolysaccharide (LPS)‐induced sepsis murine model.

## 2 MATERIALS AND METHODS

### 2.1 Animals

C57BL/6 mice with the age of 8 weeks were purchased from Nanjing University. R26Stop^FL^MEK1DD (Map2κ1*) mice were from the Jackson Laboratory. A total of 0.1 μg LPS together with 0.5 mg/g d‐galactosamine (Sigma) were injected intraperitoneally into mice for inducing sepsis. LPS (*Escherichia coli* 055:B5) were purchased from Sigma‐Aldrich. Survival was monitored for a consecutive 24 h and blood was harvested at different time points. Rigosertib was obtained from Selleck Chemicals LLC and dissolved in dimethyl sulfoxide. The stock was diluted in phosphate‐buffered saline (PBS) to the desired concentration. Each mouse was treated with 10 mg/kg rigosertib through intravenous injection. The study was approved by the Ethical Committee of the Affiliated Hospital of Jiangnan University.

### 2.2 Flow cytometry

Single‐cell suspension of the spleen in staining buffer (2% fetal bovine serum [FBS] in PBS) was stained with fluorescence‐labeled anti‐F4/80, anti‐CD19, anti‐CD3, and anti‐CD11b (eBiosciences). After wash, cells were analyzed in CytoFlex. FlowJo software was used for data analysis.

### 2.3 Preparation of bone marrow–derived macrophage

Bone marrow cells were cultured in Dulbecco's modified Eagle's medium supplemented with 10% FBS, 100 IU/penicillin, 100 µg/ml streptomycin, and 10 ng/ml macrophage‐colony stimulating factor as described previously.^14^ After 5 days of culture, differentiated bone marrow–derived macrophages (BMDMs) were monitored by F4/80 staining. The percentage of macrophages is generally more than 95%. In certain experiments, BMDMs were pretreated with 20 nM rigosertib for 1 h as previously described.^15^, ^16^, ^17^ Then these BMDMs were further treated with 100 ng/ml LPS, 20 µg/ml poly I:C or 10 µg/ml interleukin‐4 (IL‐4) for 12 or 24 h.

#### 2.3.1 Enzyme‐linked immunosorbent assay

Mice serum and cell culture supernatant were collected for cytokine measurement. IL‐6, IL‐23, TNF‐α, and IL‐1β levels were detected by commercial enzyme‐linked immunosorbent assay kits (eBioscience) following the manufacturer's instructions.

### 2.4 Hematoxylin and eosin staining

Formalin (Sigma) fixed mice lung tissues were dehydrated and embedded. Then, the fixed tissues were cut to 5‐µm slices. Hematoxylin and eosin staining was performed (Abcam) following standard protocols.

#### 2.4.1 Reverse transcription‐polymerase chain reaction

The total RNA from BMDMs was extracted by TRIzol reagent (Sigma). Then reverse transcription was completed using the PrimeScript™ RT Reagent Kit (Takara) to obtain complementary DNA. The quantitative polymerase chain reaction (PCR) reactions were set up using iQTM SYBR Green Supermix (Bio‐Rad) and subjected to the iCycler Sequence Detection System (Bio‐Rad). Primers used for real‐time PCR included: IL‐6 forward 5′‐CTGATGCTGGTGACAACCAC‐3′, reverse 5′‐CAGACTTGCCA TTGCACAAC‐3′; IL‐23a forward 5′‐CATGCTAGCCTGGAACGCACAT‐3′, reverse 5′‐ACTGGCTGTTGT CCTT GAGTCC; TNF‐α forward 5′‐CATCTTCTCAAAATTCGAGTGA CAA‐3′, reverse 5′‐CCAGCTGCTCCTCCACTTG; Arg1 forward 5′‐CATTGGCTTGCGAG ACGTAGAC‐3′, reverse 5′‐GCTGAAGGTCTCTTCCATCACC‐3′; Mrc1 forward 5′‐GTT CACCTGGAGTGA TGGTTCTC‐3′, reverse 5′‐AGGACATGCCAGGGTCACCTTT‐3′. β‐Actin forward 5′‐GAAATCGT GCGTGACATCAA AG‐3′; reverse 5′‐TGTAGTTTCATGGA TGCCACAG‐3′. The relative expression was normalized to β‐actin expression using the 2‐∆∆Ct method.

### 2.5 Western blot

BMDMs were lysed using a Protein Extraction Kit (Abcam) for protein extraction. 20‐µg proteins were subjected to sodium dodecyl sulfate‐polyacrylamide gel electrophoresis and transfer. Membranes were blocked with 5% non‐fat milk in PBST at 4°C overnight. The next day primary antibodies diluted in 1% non‐fat milk in PBST were added for incubation for 2 h at room temperature. After wash, horseradish peroxidase‐conjugated corresponding secondary antibodies were added. Chemiluminescent substrate (Bio‐Rad) was used to visualize the immunoreactive bands. Primary antibodies used in present study were: anti‐IKBα (C‐21, 1:1000), anti‐ERK (K‐23, 1:5000), anti‐phospho‐ERK (E‐4, 1:3000), anti‐MEK1 (C‐18), anti‐JNK1 (C‐17, 1:1000), anti‐p38 (H‐147, 1:1000), anti‐phospho‐JNK (Thr180/Tyr185, 1:1,000), anti‐phosphop38 (Thr180/Tyr182, 1:1000), and anti‐phospho‐MEK1/2 (Ser217/221). The first six antibodies were purchased from Santa Cruz Biotechnology, while others were from Cell Signaling Technology.

### 2.6 Statistical analysis

Data were presented as mean ±* SEM*. Two‐tailed Student's *t*‐test was used to calculate the *p*‐value. When *p*‐value is less than .05, the statistical difference was significant. Experiments were conducted independently at least three times.

## 3 RESULTS

### 3.1 Rigosertib had no effect on innate immune cells development

**Figure 1:**
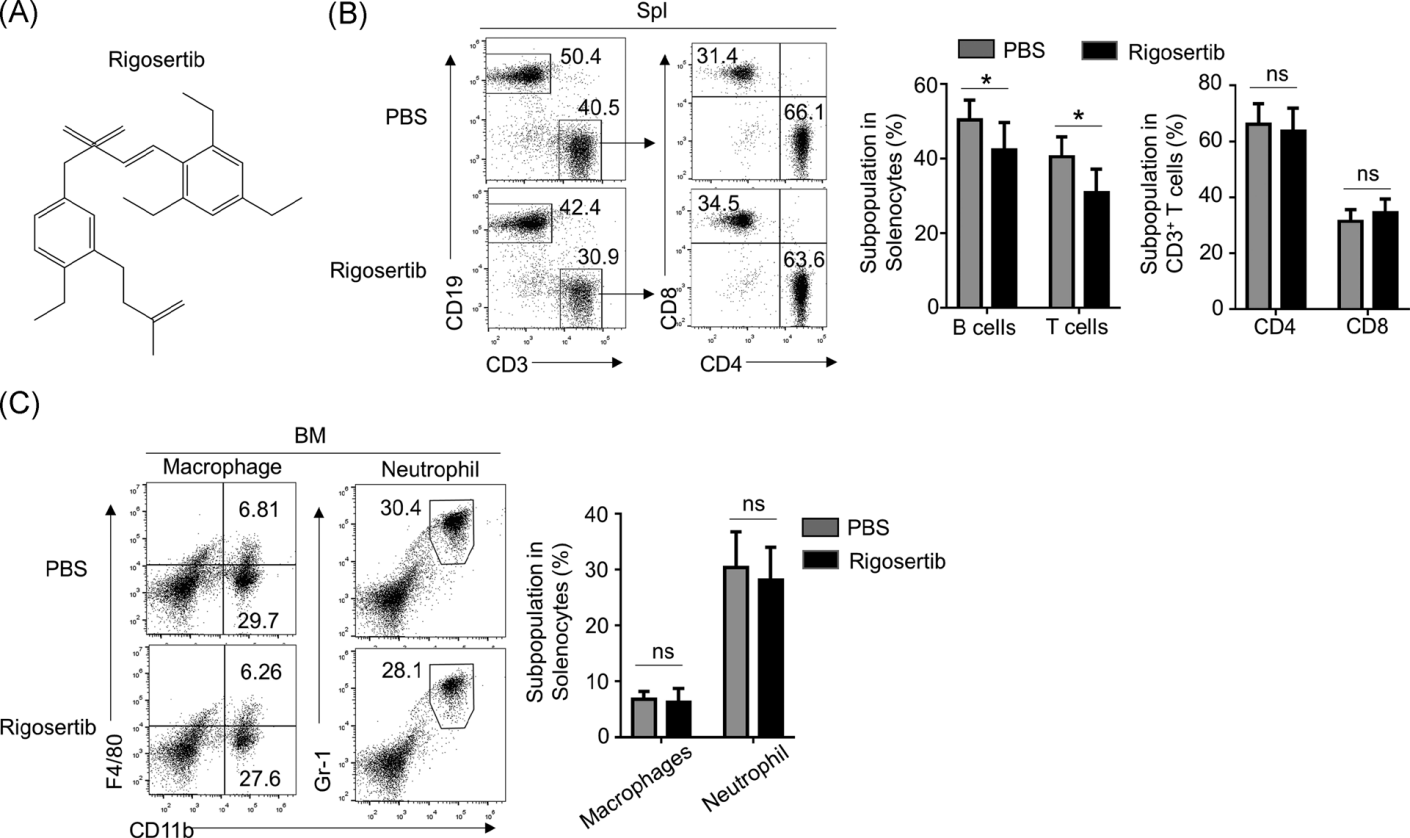
Rigosertib has no effect on the development of innate immune cells. (A) Structure of rigosertib. (B) Flow cytometry analysis of B cells (CD19^+^), T cells (CD3^+^), and subtypes of T cells in spleen from PBS‐ or rigosertib (10 mg/kg, i.v.) ‐treated mice. (C) Flow cytometry analysis of macrophages (CD11b^+^F4/80^+^) in Bone marrow from PBS‐ or Rigosertib (10 mg/kg iv) ‐treated mice. Data all panels are presented as representative FACS plots (left), and values were shown as mean ± SEM based on multiple samples (right). Similar results were obtained in three independent experiments. FACS, fluorescence‐activated cell sorting; PBS, phosphate‐buffered saline; ns, no significance

To investigate whether rigosertib (Figure 1A) affects immune cells development, we administrated mice with 10 mg/kg rigosertib or equal amount of PBS. T and B cell percentages in the spleen and the macrophages and neutrophils percentages in bone marrow were examined. As shown in Figure 1B, it was observed that PBS‐treated mice had a significantly higher percentage of CD19^+^ CD3^−^ B cells in the spleen than rigosertib‐treated mice. Similarly, PBS‐treated mice had a significantly higher percentage of CD19^−^ CD3^+^ T cells in the spleen than rigosertib‐treated mice. We further analyzed the percentage of CD4^+^ and CD8^+^ T cells in the spleen and found similar percentages of CD4^+^ T cells as well as CD8^+^ T cells in the spleen from PBS‐treated and rigosertib‐treated mice. As shown in Figure 1C, PBS‐treated mice and rigosertib‐treated mice had similar percentages of CD11b^+^F4/80^+^ macrophages as well as Gr‐1^+^CD11b^+^ neutrophils in the bone marrow. Collectively, these results indicated that rigosertib decreased T and B cells in the spleen while had no effects on the development of innate immune cells.

### 3.2 Rigosertib prevented the production of proinflammatory cytokines in LPS‐treated BMDMs

**Figure 2:**
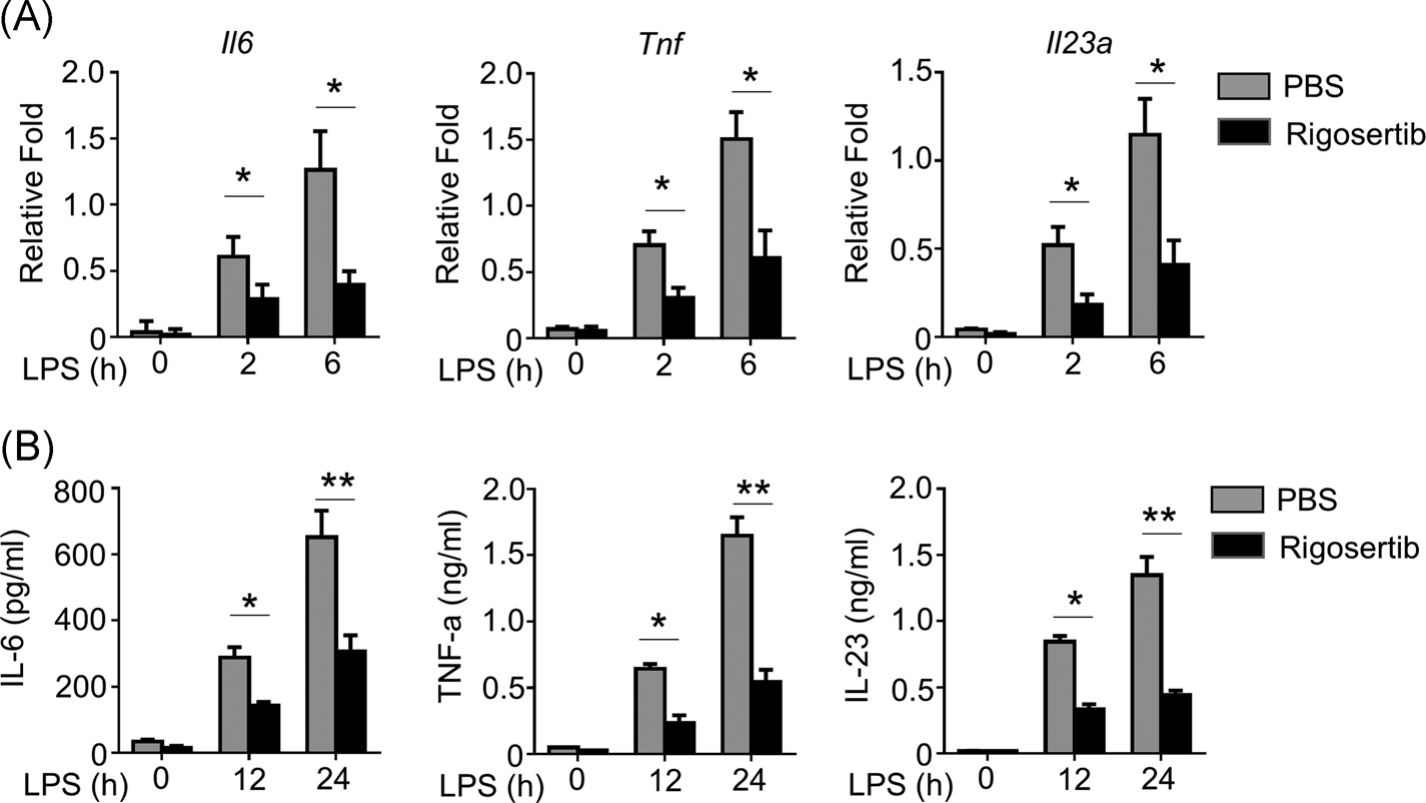
Rigosertib inhibits the production of various proinflammatory cytokines in BMDMs induced by LPS. Primary BMDMs were pretreated with 20 nM rigosertib for 1 h. (A) The expression of LPS (100 ng/ml)‐induced cytokines of rigosertib‐pretreated BMDMs were measured by qRT‐PCR. (B) ELISA of the LPS‐induced cytokines in the supernatants of rigosertib‐treated BMDMs for 12 or 24 h. All data are presented as fold relative to the Actb mRNA level. Data are shown as mean ± *SEM*. Statistical analyses represent variations in experimental replicates. BMDM, bone marrow–derived macrophage; ELISA, enzyme‐linked immunosorbent assay; IL, interleukin; LPS, lipopolysaccharide; mRNA, messenger RNA; qRT‐PCR, real‐time quantitative reverse transcription‐polymerase chain reaction; TNF‐α, tumor necrosis factor‐alpha. *p < .05, **p < .01

To evaluate the effects of rigosertib on inflammation, we administrated rigosertib to BMDMs before LPS treatment. LPS significantly increased the messenger RNA (mRNA) level of IL‐6, TNF‐α, and IL‐23a in PBS‐pretreated BMDMs, and the mRNA level increased with time increased (Figure 2A). In contrast, 2 and 6 h after LPS stimulation rigosertib‐pretreated BMDMs had significantly decreased mRNA level of IL‐6, TNF‐α, and IL‐23a when compared to PBS‐pretreated BMSMs, indicating rigosertib‐suppressed mRNA expression of IL‐6, TNF‐α, and IL‐23a after LPS stimulation. Correspondingly, we detected remarkably reduced secretion of IL‐6, TNF‐α, and IL‐23a in BMDMs pretreated with rigosertib after LPS stimulation (Figure 2B). Collectively, our results demonstrated that rigosertib prevented the expression of proinflammatory cytokines in LPS‐treated BMDMs.

### 3.3 Rigosertib suppressed the production of proinflammatory cytokines in poly I:C‐treated BMDMs

**Figure 3:**
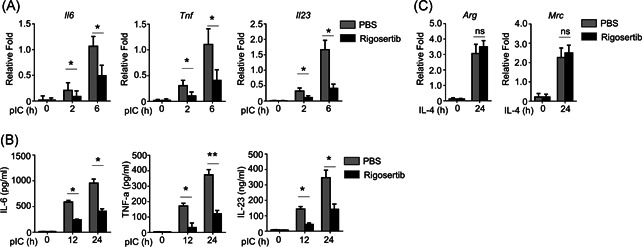
Rigosertib suppresses the poly I:C‐induced production of proinflammatory cytokines. (A) The expression of indicated cytokines induced by poly I:C (20 µg/ml) in primary BMDMs pretreated with 20 nM rigosertib for 1 h were measured by qRT‐PCR. (B) ELISA of the poly I:C ‐induced cytokines in the supernatants of rigosertib‐treated BMDMs for 12 or 24 h. (C) The expression of M2‐marker genes induced by IL‐4 (10 µg/ml) in primary BMDMs pretreated with 20 nM rigosertib for 1 h were measured by qRT‐PCR. All data are presented as fold relative to the Actb mRNA level. Data are shown as mean ± *SEM*. Statistical analyses represent variations in experimental replicates. BMDM, bone marrow–derived macrophage; ELISA, enzyme‐linked immunosorbent assay; IL, interleukin; LPS, lipopolysaccharide; mRNA, messenger RNA; qRT‐PCR, real‐time quantitative reverse transcription‐polymerase chain reaction. *p < .05, **p < .01

We continued to explore whether rigosertib inhibits the expression of proinflammatory cytokines induced by additional PAMPs in BMDMs. Similar to LPS, poly I:C induced the expression of IL‐6, TNF‐α, and IL‐23a at both mRNA and protein level in PBS‐pretreated BMDMs (Figure 3A,B) in a time‐dependent manner. Rigosertib significantly reduced the mRNA expression of IL‐6, TNF‐α, and IL‐23a in BMDMs after poly I:C stimulation. In contrast, rigosertib had no effect on the expression of Arg and Mrc, two markers of M2 macrophage (Figure 3C) in IL‐4 treated BMDMs. Collectively, our results showed that rigosertib suppressed PAMP‐induced proinflammatory cytokines production in BMDMs.

### 3.4 Rigosertib suppressed proinflammatory cytokines production by disrupting activation of the MEK1–ERK signaling axis

**Figure 4:**
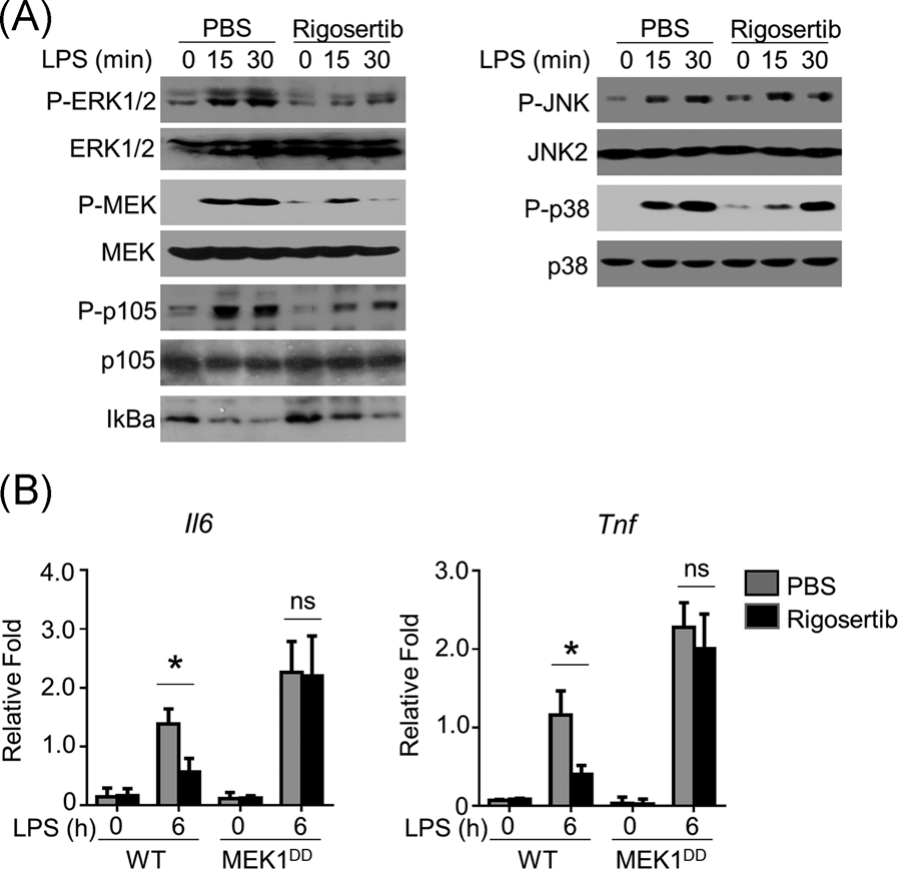
Rigosertib suppresses the LPS‐induced production of proinflammatory cytokines by disrupting the activation of MEK1–ERK axis. (A) Immunoblot analysis of the indicated phosphorylated (P‐) and total proteins in whole‐cell lysates in NF‐κB and MEK1–ERKs signal of LPS‐stimulated BMDMs that pretreated with 20 nM rigosertib for 1 h. (B) The expression of LPS‐induced cytokines in primary WT and MEK1DD BMDMs pretreated with 20 nM rigosertib for 1 h were measured by qRT‐PCR. All data are presented as fold relative to the Actb mRNA level. Data are shown as mean  ± *SEM*. Statistical analyses represent variations in experimental replicates. BMDM, bone marrow–derived macrophage; LPS, lipopolysaccharide; MEK1–ERK, extracellular signal‐regulated kinase 1/2; MAPK/ERK kinase‐1/2mRNA, messenger RNA; NF‐κB, nuclear factor‐κB; qRT‐PCR, real‐time quantitative reverse transcription‐polymerase chain reaction; WT, wild‐type. *p < .05

To examine the mechanism of rigosertib's effects on inflammation, we monitored the activation of MEK/ERK, nuclear factor‐κB (NF‐κB), and JNK2/p38 signaling pathway. LPS treatment resulted in phosphorylation of ERK1/2, MEK, p105, and degradation of IκBa, indicating LPS activated the MEK/ERK and NF‐κB signaling pathways in PBS‐treated BMDMs (Figure 4A). In contrast, rigosertib‐pretreated BMDMs had decreased protein level of p‐ERK1/2, p‐MEK at 15‐ and 30‐min after LPS treatment when compared to PBS‐pretreated BMDMs, indicating rigosertib inhibited the activation of MEK/ERK. Interestingly, rigosertib treatment did not prevent the LPS‐induced degradation of IκBa, indicating rigosertib did not prevent LPS‐induced activation of NF‐κB. We detected decreased phosphorylation of p105 in rigosertib‐pretreated BMDMs. As p105 was the target of TPL2, the upstream kinase of MEK1,^18^ the suppression of p105 phosphorylation could due to the suppression of TLP1 by rigosertib, which further indicated that rigosertib inhibited the MEK signaling pathway. In addition, rigosertib had no effect on phosphorylation of JNK2 and p38 as we detected similar protein levels of p‐JNK2 and p‐38 between PBS‐pretreated BMDMs and rigosertib‐pretreated BMDMs (Figure 4A). Collectively, these results demonstrated that rigosertib prevented the LPS‐induced activation of the MEK/ERK pathway. To confirm the participation of the MEK/ERK pathway in rigosertib‐mediated inhibition of inflammation cytokine production, we utilized BMDMs from MEK1^DD^ mice, which had inducible constitutive activation of MEK1 signaling.^19^ LPS enhanced mRNA level of IL‐6 and TNF‐α in BMDMs from wild‐type and MEK1^DD^ mice (Figure 4B). Rigosertib treatment only reduced IL‐6 and TNF‐α mRNA levels in BMDMs from wild‐type mice but not in BMDMs from MEK1^DD^ mice. Collectively, these findings demonstrated that rigosertib suppressed LPS‐induced proinflammatory cytokines expression through inhibiting MEK1–ERK activation.

### 3.5 Rigosertib ameliorated LPS‐induced sepsis by targeting MEK/ERK

**Figure 5:**
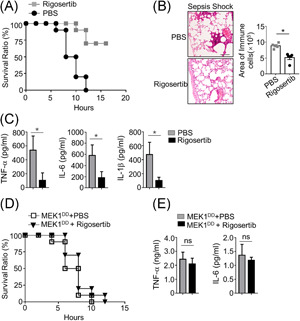
Rigosertib functions as a therapy drug for LPS‐induced sepsis shock by targeting MEK1–ERKs axis. WT mice (8‐week‐old, *n* = 10) were intravenously injected with 10 mg/kg rigosertib 2 h before injection (intraperitoneal) of LPS plus d‐galactosamine. (A) Lethality was monitored every other hour for 20 h. (B) Representative H&E slides of lung isolated from the mouse after treated with LPS for 10 h. The scale bar indicates 100 µm. (C) Mice shown were bled 10 h after injection, and the serum concentration of the indicated cytokines was determined by ELISA. A similar sepsis model was induced in MEK1^DD^ mice, which were intravenously injected with 10 mg/kg rigosertib. Lethality was monitored every other hour for 20 h (D). The serum concentration of the indicated cytokines was determined by ELISA (E). Data are shown as mean ± *SEM* values. Statistical analyses represent variations in experimental replicates. H&E, hematoxylin and eosin; IL, interleukin; ELISA, enzyme‐linked immunosorbent assay; LPS, lipopolysaccharide; MEK1–ERK, MAPK/ERK kinase‐1/2–extracellular signal‐regulated kinase 1/2; TNF‐α, tumor necrosis factor‐α; WT, wild‐type. *p < .05

To evaluate the effects of rigosertib on sepsis in vivo, we administrated rigosertib in mice 2 h before inducing the sepsis. We detected quick mice death in PBS‐treated mice after LPS treatment. In contrast, mice pretreated with rigosertib had decreased mortality when compared to PBS pretreated mice. Obvious inflammation and inflammatory cell infiltration in the lung of septic mice pretreated with PBS were detected (Figure 5B). In contrast, there was much less inflammation and inflammatory cell infiltration in the lung of septic mice pretreated with rigosertib. Moreover, septic mice pretreated with rigosertib had a significantly lower level of IL‐6, TNF‐α, and IL‐1β in serum than septic mice pretreated with PBS (Figure 5C). These findings indicated that rigosertib ameliorated LPS‐induced sepsis in mice. We continued to evaluate the effects of rigosertib on sepsis in MEK1^DD^ mice. As shown in Figure 5D, septic MEK1^DD^ mice pretreated with rigosertib had a similar survival rate to septic MEK1^DD^ mice pretreated with PBS, indicating rigosertib did not affect mortality in septic MEK1^DD^ mice. We did not detect any significant difference of serum level of IL‐6 and TNF‐α between PBS‐pretreated and rigosertib‐pretreated septic MEK1^DD^ mice. These findings proved that the effects of rigosertib were abolished in MEK1^DD^ mice, indicating rigosertib ameliorated sepsis through targeting MEK signaling pathway.

## 4 DISCUSSION

In this study, the prospective effects of rigosertib on sepsis were investigated. We found that rigosertib inhibited LPS and poly I:C‐induced expression of proinflammatory cytokine in BMDMs. Rigosertib prevented the activation of the MEK/EKR signaling pathway induced by LPS in BMDMs. We also proved that rigosertib ameliorated lung injury and promoted the survival rate of septic mice. In contrast, these protective effects were abolished in MEK1^DD^ mice which had constitutive activation of MEK signaling, indicating the protection of rigosertib against sepsis depended on inhibition of MEK/ERK signaling. Our finding suggested that rigosertib could be a potential reagent to treat sepsis.

The dysregulated immune responses during infection lead to sepsis, which involved over‐activation at an early stage and then suppression of immune response.^20^ During sepsis, the innate immune responses are activated by PAMPs/PRR at the early stage, which initiates overwhelming inflammatory response including producing overwhelming proinflammatory cytokines. These cytokines further launch and escalate innate and adaptive immune responses and finally lead to tissue damage.^21^ Upon stimulation, macrophages are activated and produced an abundant amount of proinflammatory cytokines including TNF‐α, IL‐1β, and IL‐6. TNF‐α, IL‐1β, and IL‐6 are critical cytokines during infection and elevated levels of these cytokines are associated with sepsis.^22^, ^23^ On other hand, inhibition of these cytokines by antagonists, neutralization antibodies, and specific inhibitors reduces the inflammatory response and ameliorates symptoms in both experimental animals and clinical patients,^24^, ^25^, ^26^, ^27^ indicating targeting inflammatory cytokines could treat sepsis.

In sepsis, various MAPK signaling networks are activated, thereby contributing to transcription of proinflammatory factors which are critical in sepsis.^28^ Inhibition of MAPK signaling pathway could suppress proinflammatory cytokines production. For example, berberine suppressed the activation/phosphorylation of p38, JNK, and ERK and suppressed the expression of IL‑1β, IL‐6 in LPS‐stimulated macrophages.^29^, ^30^ Hesperidin inhibited LPS‑induced activation of JNK and p38 MAPK pathways and prevented LPS‐induced endotoxicity in rats.^31^ JNK and p38 MAPK inhibitor significantly reduced IL‑6 and TNF‑α expression and attenuated acute lung injury in septic rats.^32^ Therefore, targeting MAPK signaling pathways should be an effective approach to treat sepsis.

Rigosertib is a multikinase inhibitor and a selective anticancer agent, which has been shown to inhibit Polo‐like kinase 1 (PLK1) and phosphatidylinositol 3‑kinase/protein kinase B (PI3K/Akt) pathway.^11^, ^17^ These inhibitory activities of rigosertib have been evaluated in clinical trials against multiple cancers.^33^ The regulatory effects of rigosertib on inflammation have also been described. Using the induced colitis mice model, Rahmani et al.^11^ reported that rigosertib suppressed the production of IL‐1β, TNF‐α, INF‐γ, and MCP‐1 through PI3K/AKT and NF‐κB signaling pathways and protected mice against colitis. Interestingly, RLK1, the substrate of rigosertib, has been shown to be involved inflammation. Hu et al.^34^ described that inhibiting PLK1 resulted in downregulation of activation of ERK, p38, and NF‐κB induced by LPS and Pam3CSK4. The inhibition of RLK1 by rigosertib could contribute to rigosertib's anti‐inflammatory activity.

In the present study, we demonstrated that in septic mice, rigosertib‐suppressed inflammation through inhibiting MEK/EKR pathway. Classic ERK1/2 activation is initiated by binding of VEGF, IGF‐1, EGF to their respective receptor tyrosine kinases causing the activation of the Ras/Raf/MEK/ERK pathway.^35^ However, increasing evidence indicates that TLRs can also activate ERK1/2. TLR4 stimulation by LPS increases ERK1/2 activity in macrophages by engaging the tumor progression locus‐2 (TPL‐2)/MEK1/2/ERK1/2 cascade.^36^ Our resulted demonstrated that rigosertib prevented the phosphorylation of p105, a substrate of TPL‐2, suggesting rigosertib inhibited the LPS/TLR4‐induced production of inflammatory cytokines by inhibiting TPL‐2/MEK/ERK cascade. A recent study demonstrated rigosertib mimicked RAS, disrupt the activation of RAF, and inhibited the RAS‐RAF‐MEK pathway.^10^ There was no direct evidence in our present study to show the involvement of RAS and RAF in rigosertib‐regulated inhibition of MEK/ERK activation. Patriotis et al.^37^ reported that TPL‐2 may participate in RAS/RAF complex assembling, which was required for activation of downstream MEK. Therefore, it would be interesting and necessary to explore the potential role of TPL‐2, RAS, and RAF in rigosertib‐mediated amelioration of inflammation in sepsis. In the present study, we administrated rigosertib to mice by intravenous injection. However, in current clinical trials, rigosertib is administrated orally. The biosafety, side effects of intravenous injection of rigosertib remain unknown. In addition, it is unclear that whether oral administration of rigosertib could have similar protective effects on sepsis in mice. Experiments need to be performed to address these concerns. Rigosertib alleviated LPS‐induced sepsis inhibits MEK1/ERK signaling pathway.

## CONFLICT OF INTERESTS

The authors declare that there are no conflict of interests.

## ETHICS STATEMENT

The study was approved by the Ethics Committee of the Affiliated Hospital of Jiangnan University and followed Guide for the Care and Use of Laboratory Animals (Eighth edition, NIH).

## Data Availability

Data is available from the authors by request.
